# Is an Early Resumption of a Regular Diet After Laparoscopic Roux-en-Y Gastric Bypass Safe?

**DOI:** 10.1007/s11695-022-06224-4

**Published:** 2022-08-01

**Authors:** Mickael Chevallay, Eleftherios Gialamas, Guillaume Giudicelli, Aurélie Vuagniaux, Laetitia Guarino, Marc Worreth, Stéphane Saillant, Michele Diana, Alend Saadi

**Affiliations:** 1 Surgery Department, Neuchâtel Hospital, 2000 Neuchâtel, Switzerland; 2 Unit of Visceral Surgery, Department of Surgery, Geneva University Hospital, 1205 Geneva, Switzerland; 3 Center for Psychiatric Emergencies and Liaison Psychiatry, Department of General and Liaison Psychiatry, Neuchâtel Psychiatry Center, 2000 Neuchâtel, Switzerland; 4 Surgery Department, Strasbourg University Hospital, 67000 Strasbourg, France; 5 IRCAD, Research Institute Against Digestive Cancer, 67091 Strasbourg, France

**Keywords:** Postoperative care, Obesity, Bariatric surgery

## Abstract

**Background:**

Return to a normal diet is a crucial step after bariatric surgery. Proximal anastomosis is a source of concern for early feeding as the passage of solid food through a recent anastomosis could well increase pressure and the risk of leakage. This study aims to assess the safety of an early normal diet after a laparoscopic Roux-en-Y gastric bypass (LRYGB).

**Materials and Methods:**

All consecutive patients undergoing primary LRYGB between January 2015 and December 2020 were included prospectively. Three postoperative pureed diets were compared at 4 weeks, 2 weeks, and 1 week. All-cause morbidity at 90 days was the main outcome. Overall complications, severe complications (Clavien-Dindo ≥ grade 3a), length of hospital stay, number of emergency, and unplanned consultations during the 3 postoperative months were recorded for each group.

**Results:**

Three hundred and sixty-seven patients with a mean BMI of 42.10 kg/m^2^ (± SD: 4.78) were included. All-cause morbidity at 90 days was 11.7% (43/367) and no significant difference was observed between the 3 groups. Adjustment for patients and operative cofounders did not demonstrate any increased risk of postoperative complications between the 3 groups, with an odds ratio of 1, 1.23(95% CI [0. 55–2.75]), and 1.14 (95% CI [0.49, 2.67]) for groups 1, 2, and 3 respectively. Severe complications (Clavien-Dindo ≥ grade 3a) and emergency or unplanned consultations were also similar in the 3 groups.

**Conclusion:**

Return to a normal diet 1 week after LRYGB did not increase short-term morbidity and unplanned consultations. It may be safe and contribute to patient comfort.

**Graphical abstract:**

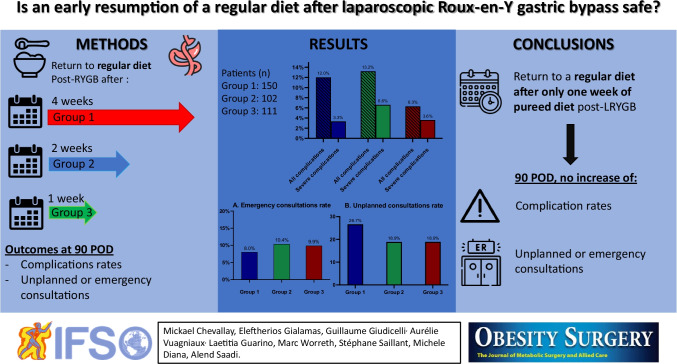

## Introduction

Morbid obesity is a major public health burden in developed countries (1). The only effective long-term treatment for severe obesity is bariatric surgery (2). Laparoscopic Roux-en-Y gastric bypass (LRYGB) is the second most performed bariatric surgery worldwide and the most performed in Switzerland with 80% of cases in 2020 (3, 4). LRYGB includes the creation of a small gastric pouch of 25 to 50 mL with a gastrojejunal anastomosis. The food bolus distending this proximal anastomosis may be a source of concern. On the other hand, enteral feeding has been proven to promote mucosal growth, increase gastrointestinal immunity, and decrease mucosal permeability (5, 6). Early postoperative normal feeding after upper gastrointestinal (UGI) surgery causes a dilemma, namely a rapid return to a normal diet to promote patient recovery but slow enough to allow anastomotic healing.

Early feeding has been proven to be safe after small bowel resection (7), lower gastrointestinal surgery (8), and gastric cancer surgery (9). For bariatric surgery, there is a lack of consensus regarding the postoperative regimen composition. The European guidelines for bariatric surgery recommend a texture progression specific to the surgical procedure and to the bariatric center’s usual practice (10). On the other hand, the US guidelines propose a return to normal food intake initiated between 4 and 6 weeks after surgery (11). In practice, it results in a liquid diet and then possibly to a pureed diet during the entire period. However, in the current context of fast-track surgery, there is a growing interest in early normal food intake after abdominal surgery, allowing for a shorter hospital stay and even for outpatient surgery. The timing to introduce a normal diet is a matter of concern as high-quality research evidence is scarce. A strict diet may impede patient quality of life, patient compliance, and it may also induce nutritional deficiencies (12, 13). Patient quality of life and satisfaction are significant aspects of the postoperative period, which are increasingly studied (14). Patients prioritize quality-of-life items much more often than healthcare professionals (15). Bariatric surgeons might be too strict with their postoperative diet recommendations. The aim of the study was to investigate the safety of introducing a normal textured diet 1 week after LRGYB.

## Materials and Methods

### Study Population and Design

All consecutive adult patients undergoing a primary LRYGB at our institution were prospectively included from the start of the bariatric program in January 2015 until December 2020. All patients met the International Federation of Surgery for Obesity (IFSO) criteria for bariatric surgery (16). Patients undergoing other bariatric procedures (e.g., gastric band, laparoscopic sleeve gastrectomy, conversion of laparoscopic sleeve gastrectomy to a Roux-en-Y gastric bypass), and patients undergoing associated surgical procedures, except for cholecystectomy, were excluded.

A comparison of perioperative outcomes was performed for all patients according to 3 different durations of strictly pureed postoperative diets. Patients operated on from January 2015 to August 2017 were recommended to take a pureed diet of 4 weeks (group 1), from September 2017 to February 2019, namely 2 weeks (group 2), and from March 2019 to January 2020, namely 1 week (group 3). Patients were allowed to resume normal food texture intake afterwards.

All-cause postoperative morbidity at 90 days was classified according to the Clavien-Dindo classification (17). All patient emergency or unplanned consultations (with a dietician or a surgeon) and readmission during the 90 postoperative days were monitored prospectively. Other secondary outcomes included mortality, severe complications (Clavien-Dindo ≥ grade 3a), and length of hospital stay.

We documented patient demographics, obesity-related-associated medical conditions, body mass index (BMI), operative time, anastomotic techniques (i.e., hand-sewn, mechanical, or mixed when one anastomosis was hand-sewn and the other one was mechanical), adverse intraoperative events (i.e., any unexpected one occurring during the procedure, and for instance, hemorrhage, viscus perforation, anastomotic oversuturing), simultaneous cholecystectomy, hiatus reconstruction, as well as intensive care unit (ICU) admission.

### Surgical Technique

The surgical approach was described in our previous article (18).

All patients were screened preoperatively by a multidisciplinary bariatric team, and written informed consent was obtained. Esophagogastroduodenoscopy, assessment for sleep apnea, and gallstones using ultrasonography were performed. Patients with gallstones underwent a concomitant cholecystectomy.

Of interest, our anastomotic technique changed over time, going from linear stapling to totally hand-sewn anastomosis. This change reflects a modification in the surgical habits over the period of the study. In the mechanical anastomotic technique, an antegastric end-to-side 3-cm gastrojejunostomy (GJ) anastomosis was created with a 45-mm linear stapler and the stapler opening was closed by means of a STRATAFIX™ (Ethicon Endo-Surgery, Inc., Cincinnati, OH, USA) running suture. In the hand-sewn anastomotic technique, an end-to-side GJ anastomosis of 2 cm in diameter was created with two STRATAFIX ™ full-thickness running sutures.

All patients received subcutaneous thromboprophylaxis with low-molecular-weight-heparin (LMWH) the day before and 6 h after surgery, according to their body weight and until 30 days after discharge. We allowed free liquid intake on postoperative day 0 and introduced a pureed diet on postoperative day 1. After adequate liquid intake and pain control, patients were discharged home.

### Diet Recommendation

**Fig. 1:**
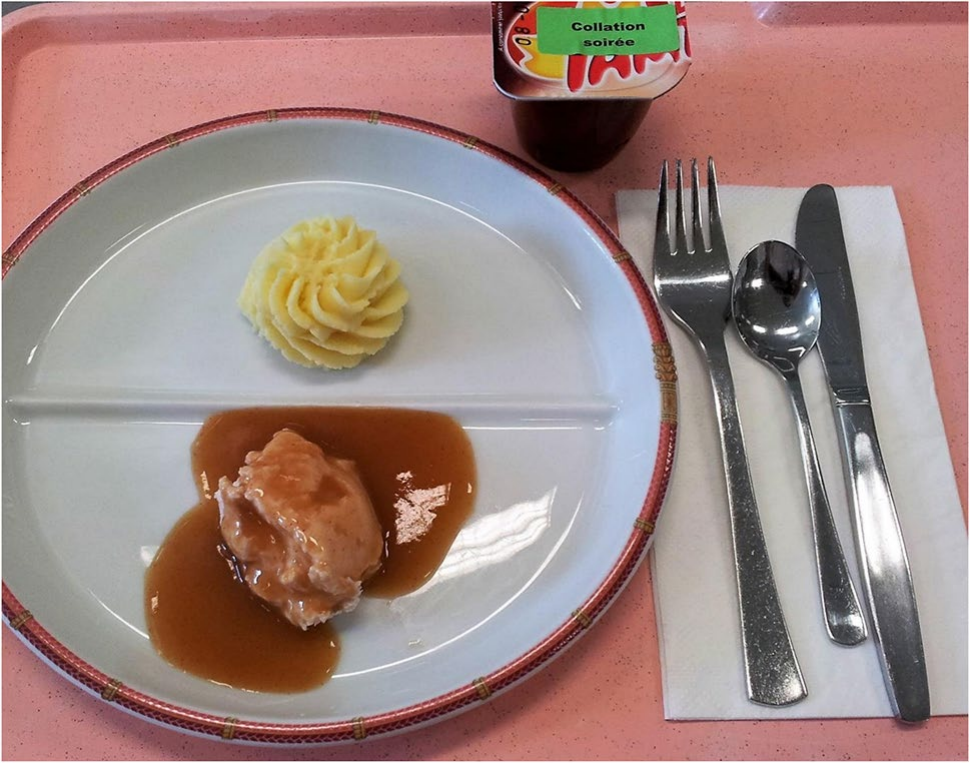
Postoperative day one example of a typical pureed diet meal tray. At the top of the picture: chocolate flan. In the middle: mashed potatoes. Bottom of the picture: pureed chicken meat

To address patient compliance to the postoperative pureed diet, all patients had two specific preoperative consultations with a dietician, with explanations regarding the content of the postoperative diet, as well as its duration. Exceptionally, if a patient was unable to prepare a pureed diet, home-delivered pureed food was organized in the postoperative period. On the first postoperative day, a dietician was present for the first meal. Patients were then regularly followed up by the dietician’s team and patient adherence to the pureed diet was reassessed during these consultations. Patients were advised to eat at least 3 meals a day with a duration of 30 min allocated for each meal. The meal should contain at least 60 g of protein. To prevent dehydration, patients were advised to drink 1.5 L of water and to stop drinking within 30 min of mealtimes. The definition of pureed diet involved the mechanical alteration of the consistency of food, in order for it not to require any chewing (19). Figure 1 shows a representative first day example of a typical pureed diet meal tray. The first follow-up appointment was scheduled for 7 to 10 days following patient discharge.

### Statistical Analysis

We compared the 3 time periods in terms of patient characteristics, operation characteristics, and patient outcomes. A chi-square test was used for categorical variables and analysis of variance (ANOVA) for comparisons of means. The significance threshold was set with a *p* value inferior to 0.05. We examined the risk of severe postoperative complications according to patient and operation characteristics. A logistic regression model was created to describe the effect of the period after adjustment for possible confounders. We built an unadjusted model, a second model adjusted only for patient characteristics which were associated with the risk of complication (age ≥ 50 years, BMI ≥ 40, and diabetes mellitus), a third model adjusted only for operation characteristics (duration of surgery ≥ 90 min and occurrence of adverse events during surgery), and a fourth model adjusted for all potential confounders. The analysis was performed using the IBM SPSS statistics Base version 20.0 (IBM Corp. Released 2011. IBM SPSS Statistics for Windows, Version 20.0. Armonk, NY: IBM Corp.).

## Results

### Overall Period Characteristics

We included a total of 367 patients. The mean length of hospital stay was 2.49 days (± SD 1.147). Overall postoperative complications occurred in 43 patients (11.7%). A deep surgical site infection was observed in 10 patients (2.72%). In 6 patients (1.6%), there was a leakage (anastomotic or small bowel perforation) requiring radiologic or surgical interventions. Table 1 summarizes the etiology and treatment for each of these patients. Pulmonary embolism was noted in 1 patient (0.3%) and anastomotic bleeding in 12 patients (3.26%). One patient died during the in-hospital period. The overall mortality rate was 0.3%. Severe complications (Clavien-Dindo ≥ grade 3a) were observed in 16 patients (4.4%). Readmissions were noted in 18 patients (4.9%) with a mean time after surgery to readmission of 6.22 days (± SD 2.96). During the 3 postoperative months after hospital discharge, emergency consultations were observed in 34 patients (9.3%). Besides the 2 planned postoperative consultations with a dietician during the 30 postoperative days, an additional consultation was requested by 81 patients. The mean number of days between patient discharge and the emergency consultation was 22.47 days (± SD 23.68).

**Table 1:** Description and treatment of patients with leakage or suspected leakage requiring endoscopic, radiologic, or surgical interventions. JJA, jejunojejunal anastomosis; GJA, gastrojejunal anastomosis; CD, Dindo-Clavien classification

Patient *N*°	Group *N*°	Age	Etiology	Leakage diagnosis	Leakage treatment	CD
1	1	49	Kinking of JJA	Intraoperative	GJA reinforcement with sutures	IIIb
2	2	45	Pressure from a hematoma originating from the excluded stomach staple line bleeding	Intraoperative	GJA reinforcement with sutures	IVb
3	2	45	Perforation on a small bowel lesion	Intraoperative	Suture of the lesion	IIIb
4	3	58	Early small bowel ileus incarcerated in a trocar introduction site	Intraoperative	Reconstruction of JJA and GIA	V
5	3	20	Small bowel ileus caused by an early adhesion	Intraoperative	GJA reinforcement with sutures	IIIb
6	3	42	Small abscess near the JJA	Suspected on CT-scan but not cofirmed intraoperatively	drainage	IIIb

## Period Comparison for 3 Pureed Diets

One hundred and fifty patients (40.88%) were operated on during the 4-week pureed postoperative diet period (group 1), 106 (28.88%) during the 2-week pureed postoperative diet period (group 2), and 111 (30.24%) during the 1-week pureed postoperative diet period (group 3). The results are summarized in Tables 2 and 3.

**Table 2:** Patient and intraoperative characteristics in each group of diet recommendations (group 1: 4 weeks, group 2: 2 weeks, group 3: 1 week)

	Group 1 *N* = 150	Group 2 *N* = 106	Group 3 *N* = 111	*p* value
Women, *N* (%)	123 (82.0)	88 (83.0)	95 (85.6)	0.74
Age, mean (SD)	41.6 (11.3)	42.8 (11.4)	43.1 (11.4)	0.51
Body mass index (BMI), mean standard deviation (SD)	41.3 (3.8)	42.4 (5.0)	42.8 (6.7)	0.034
Severe obesity (BMI ≥ 40), *N* (%)	98 (65.3)	70 (66.0)	78 (70.3)	0.68
High blood pressure, *N* (%)	47 (31.3)	26 (24.5)	38 (34.2)	0.28
Diabetes mellitus, *N* (%)	29 (19.3)	25 (23.6)	11 (9.9)	0.025
Chronic obstructive pulmonary disease (COPD), *N* (%)	1 (0.7)	0 (0.0)	5 (4.5)	0.016
Obstructive sleep apnea syndrome (OSAS), *N* (%)	81 (54.0)	48 (45.3)	57 (51.4)	0.38
Hiatal hernia, *N* (%)	29 (19.3)	26 (24.5)	21 (18.9)	0.51
Duration of surgery, minutes, mean (SD)	113 (34)	90 (30)	89 (25)	< 0.001
Type of anastomosis, *N* (%) - Mechanical - Hand-sewn - Mixed	148 (98.0) 0 (0.0) 2 (2)	98 (92.5) 0 (0.0) 8 (7.5)	21 (18.9) 72 (64.9) 18 (16.2.0)	< 0.001
Adhesiolysis, *N* (%)	7 (4.7)	4 (3.8)	5 (4.5)	0.94
Cholecystectomy (CCK), *N* (%)	20 (13.3)	18 (17.0)	9 (8.1)	0.14
Hiatus reconstruction, *N* (%)	25 (16.7)	10 (9.4)	9 (8.1)	0.07
Complication during surgery, *N* (%)	2 (1.3)	6 (5.7)	0 (0.0)	0.011

**Table 3:** Postoperative outcomes (group 1: 4 weeks, group 2: 2 weeks, group 3: 1 week)

	Group 1 *N* = 150	Group 2 *N* = 106	Group 3 *N* = 111	*p* value
Overall complications, *N* (%)	18 (12.0)	14 (13.2)	11 (9.9)	0.75
Severe complications (> Dindo-Clavien grade 3a), *N* (%)	5 (3.3)	7 (6.6)	4 (3.6)	0.40
Leakage (anastomotic or small bowel perforation), *N* (%)	1 (0.6)	2 (1.9)	3 (2.7)	0.42
Hospital length of stay, mean (SD)	3.1 (0.8)	2.3 (1.5)	1.9 (0.7)	< 0.001*
Unplanned consultations, *N* (%)	40 (26.7)	20 (18.9)	21 (18.9)	0.43
Readmissions, *N* (%)	6 (4.0)	5 (4.7)	7 (6.3)	0.69
Emergency consultations, *N* (%)	12 (8.0)	11 (10.4)	11 (9.9)	0.78
Cause of emergency consultations				
Abdominal pain	26 (17.3)	12 (11.3)	11 (9.9)	0.17
Dumping syndrome	15 (10.0)	5 (4.7)	5 (4.5)	0.13
Nausea	2 (1.3)	2 (1.9)	3 (2.7)	0.73
Reflux	7 (4.7)	3 (2.8)	1 (0.9)	0.21

**Fig. 2:**
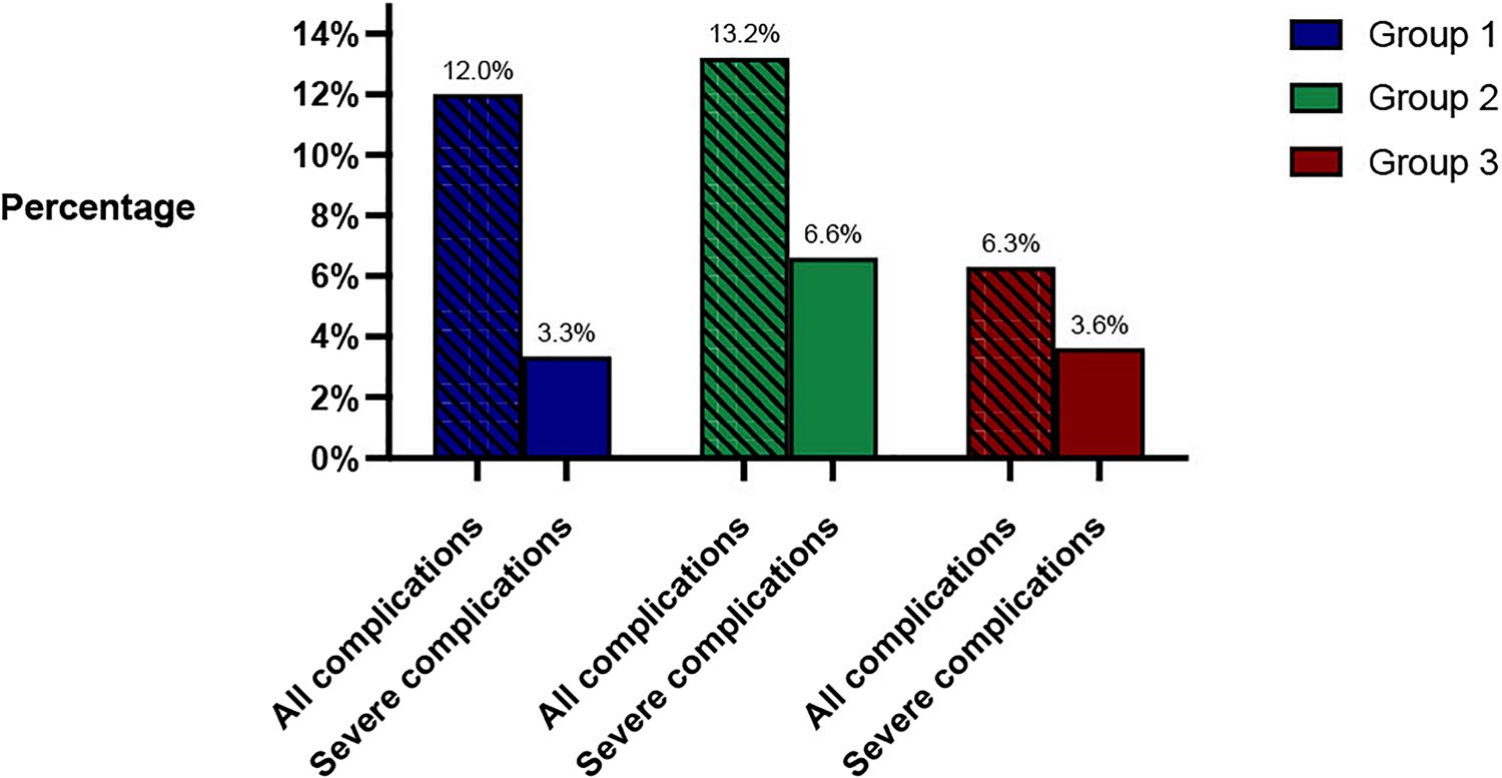
Rate of overall and severe complications for each group of diet recommendations (group 1: 4 weeks, group 2: 2 weeks, group 3: 1 week)

The overall 90-day complication rate was 12% in group 1, 13.2% in group 2, and 9.9% in group 3 with no statistically significant difference. The clinical follow-up rate was 100% at 90 days following the LRGYB intervention. Figure 2 shows the rate of overall and severe complications of the 3 groups.

### Unplanned and Emergency Consultations

**Fig. 3:**
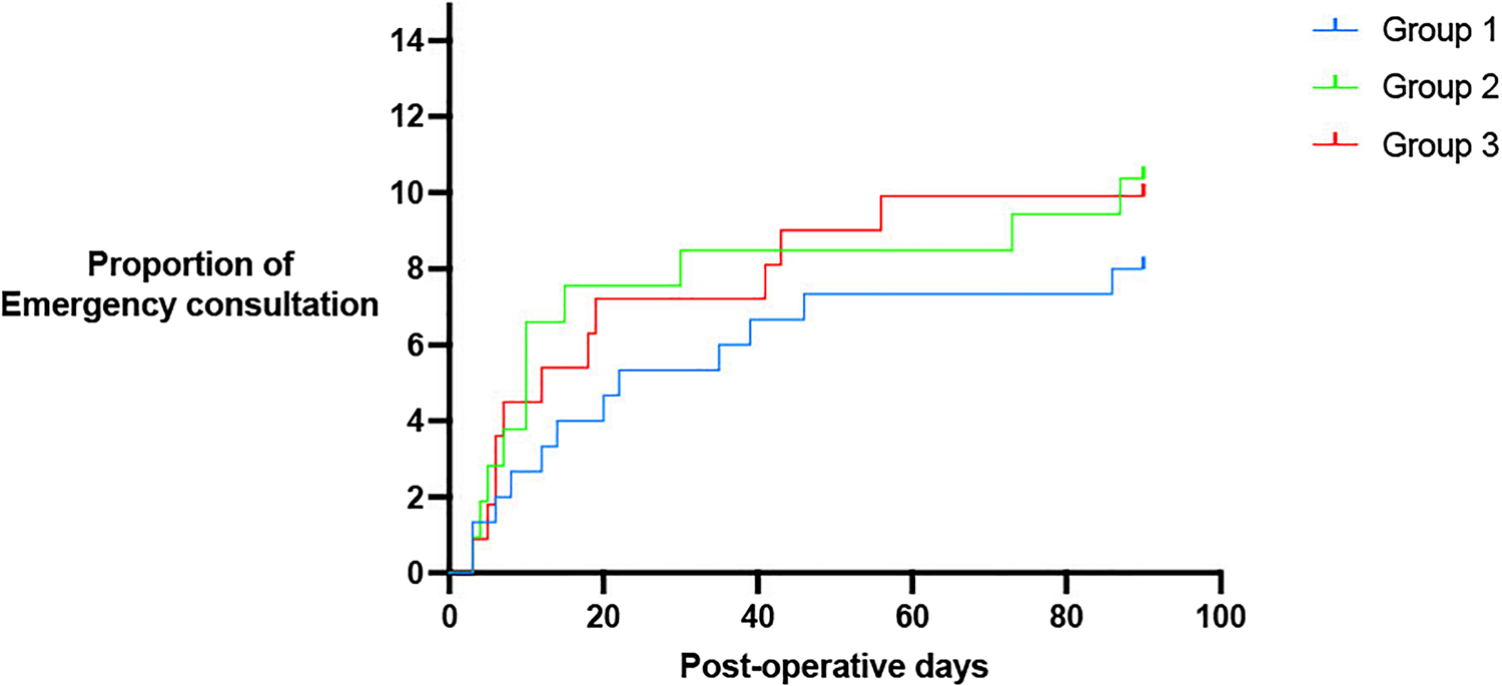
Kaplan–Meier analysis for the proportion of emergency consultations for LRYGB patients according to their diet recommendations (group 1: 4 weeks, group 2: 2 weeks, group 3: 1 week)

**Fig. 4:**
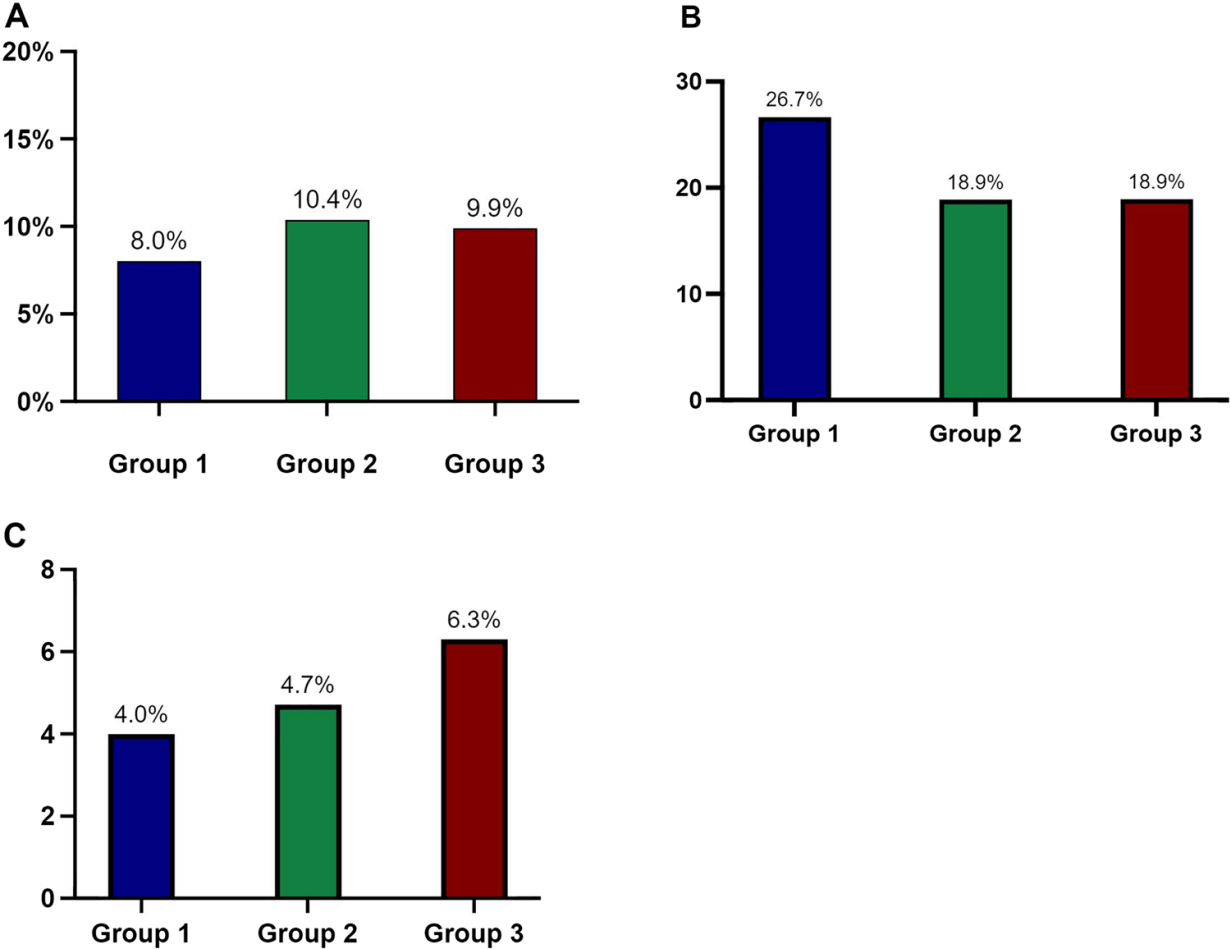
Rates for each group of diet recommendations of **A** emergency consultations, **B** unplanned consultations with a dietician or a surgeon, and **C** readmissions at 90 days (group 1: 4 weeks, group 2: 2 weeks, group 3: 1 week)

No difference related to emergency consultations was noted between the 3 groups (Table 3). Figure 3 shows the Kaplan–Meier analysis for emergency consultation probability during the 90-day postoperative period between the 3 groups. Figure 4 illustrates the emergency, unplanned, and readmission rates for each group.

### Multivariate Analysis for Overall Complications

Tables 4 and 5 summarize patient and operative characteristics associated with complications. No patient characteristic was associated with a higher risk of complications. In operative characteristics, surgical time, intraoperative adverse event, and hiatus reconstruction were associated with a higher rate of severe complications.

**Table 4:** Complication rates depending on patient characteristics

	Overall complications	*p* value	Severe complications	*p* value
Gender		0.71		0.25
Women	35 (11.4)		15 (4.9)	
Men	8 (13.1)		1 (1.6)	
Age (years)		0.57		0.29
19–29	7 (11.7)		4 (6.7)	
30–39	8 (9.8)		1 (1.2)	
40–49	11 (9.7)		7 (6.2)	
50–70	17 (15.2)		4 (3.6)	
BMI		0.27		0.49
30–40	11 (9.1)		4 (3.3)	
≥ 40	32 (13.0)		12 (4.9)	
High blood pressure		0.72		0.31
No	31 (12.1)		13 (5.1)	
Yes	12 (10.8)		3 (2.7)	
Diabetes mellitus		0.062		0.91
No	31 (10.3)		13 (4.3)	
Yes	12 (18.5)		3 (4.6)	
COPD		0.37		0.60
No	43 (11.0)		16 (4.4)	
Yes	0		0	
OSAS		0.091		0.33
No	16 (8.8)		6 (3.3)	
Yes	27 (14.5)		10 (5.4)	
Hiatal hernia		0.66		0.29
No	33 (11.3)		11 (3.8)	
Yes	10 (13.2)		5 (6.6)

**Table 5:** Difference in the rate of overall and severe complications according to surgical confounders

	Overall complications	*p* value	Severe complications	*p* value
Surgery time		0.033*		0.92
48–59 min	1 (7.1)		1 (7.1)	
60–89 min	10 (6.8)		7 (4.8)	
90–119 min	22 (18.3)		5 (4.2)	
120–240 min	10 (11.5)		3 (3.4)	
Type of anastomosis, *N* (%)		0.42		0.83
- Mechanical	32 (12.0)		13 (4.9)	
- Hand-sewn	10 (13.9)		3 (4.2)	
- Mixed	1 (20.0)		0	
Adhesiolysis		0.14		0.38
No	43 (12.3)		16 (4.6)	
Yes	0		0	
Cholecystectomy		0.09		0.14
No	34 (10.6)		12 (3.8)	
Yes	9 (19.1)		4 (8.5)	
Intraoperative adverse event		0.022*		< 0.001*
No	40 (11.1)		13 (3.6)	
Yes	3 (37.5)		3 (37.7)	
Hiatus reconstruction		0.16		0.015*
No	35 (10.8)		11 (3.4)	
Yes	8 (18.2)		5 (11.4)	

Using these 2 tables, we selected potential confounders with patient characteristics (age > 50 years, BMI > 40, presence of diabetes mellitus, history of obstructive sleep apnea) and operative characteristics such as operative time superior to 90 min, increase in technical aspects (cholecystectomy or hiatal reconstruction), and intraoperative adverse events.

After adjustments for patient characteristics and operative characteristics, there was no increased risk for overall postoperative complications between the 3 groups (odds ratio: group 1 = 1, group 2 = 1.23, 95% CI (0. 55, 2.75), and group 3 = 1.14, 95% CI (0.49, 2.67)).

Table 6 summarizes the odds ratio for overall postoperative complications with the different adjustment models.

**Table 6:** Odds ratio for 90-day postoperative complications according to diet recommendation periods (unadjusted, adjusted for patient characteristics, adjusted for operation characteristics, and adjusted for both) (OR, odds ratio; CI, confidence interval; BMI, body mass index)

	Unadjusted		Adjusted for patient characteristics		Adjusted for operation characteristics		Adjusted for all variables	
	OR (95% CI)	*p*	OR (95% CI)	*p*	OR (95% CI)	*p*	OR (95% CI)	*p*
Group 1 Group 2 Group 3	1.0 1.12 (0.53, 2.36) 0.81 (0.36, 1.78)	0.77 0.60	1.0 1.10 (0.52, 2.34) 0.82 (0.37, 1.86)	0.77 0.62	1.0 1.23 (0.56, 2.73) 1.11 (0.48, 2.54)	0.61 0.80	1.0 1.23 (0. 55, 2.75) 1.14 (0.49, 2.67)	0.62 0.76
Age ≥ 50 years	-		1.35 (0.67, 2.73)	0.40	-		1.08 (0.52, 2.23)	0.81
BMI ≥ 40	-		1.45 (0.69, 3.05)	0.33	-		1.36 (0.63, 2.94)	0.43
Diabetes mellitus	-		1.75 (0.82, 3.72)	0.15	-		1.57 (0.72, 3.44)	0.26
Sleep apnea	-		1.53 (0.71, 2.91)	0.32	-		1.63 (0.79, 3.38)	0.19
Operative time ≥ 90 min	-		-		2.20 (1.03, 4.72)	0.042	2.02 (0.93, 4.40)	0.076
Hiatus reconstruction	-		-		1.68 (0.70, 3.99)	0.24	1.83 (0.76, 4.40)	0.18
Intraoperative adverse event	-		-		3.31 (0.70, 15.53)	0.13	3.27 (0.67, 15.91)	0.14

## Discussion

This retrospective study compared the short-term outcomes of different postoperative nutritional recommendations. A return to a normal diet 1 week after LRYGB did not increase the rate of postoperative complications or unplanned consultations at 90 days.

Timing of normal feeding after upper gastrointestinal surgery is a subject of uncertainty. In oncological situations, after esophagectomy and gastrectomy, several studies assessed the safety of early pureed oral feeding. Hur et al. (20) included 58 patients with total and subtotal gastrectomy for gastric cancer. Patients were randomized between the early feeding group with a pureed diet on postoperative day 3 (POD) and a control group with a pureed diet on postoperative day 6, and no difference in postoperative morbidity or reoperation rates was observed. Six randomized studies were included by Liu et al. (21) in a meta-analysis and confirmed the safety of early oral feeding, defined as feeding initiated before the first flatus was passed, after gastric cancer. There was no difference in terms of postoperative complications, tolerability of oral feeding, and readmission rate. For esophagectomy, a randomized trial from Sun et al. (22) assessed the complications rates in patients after a McKeown minimally invasive esophagectomy for esophageal cancer. Patients were randomized between the ones who received soft solid food on the first postoperative day (POD) or on POD 7. One hundred and forty patients were enrolled in each group. There was no difference in cardiac, respiratory, or gastrointestinal complications (25% in the early feeding group versus 27.9% in the late feeding group). Quality of life was improved in the early feeding group. Even in cervical anastomoses, early feeding does not seem to put reconstruction at risk.

For LRYGB, the safety of early oral intakes was studied by Bevilacqua et al. (23). In their retrospective study, they included 244 patients who underwent a laparoscopic sleeve gastrectomy and LRYGB. A change in their postoperative diet protocol was implemented with a full liquid diet on POD 0 as compared to the previous POD 1 in their previous protocol. There was no difference in postoperative complications between the 2 groups. The length of hospital stay was shorter in the early feeding group (36.2 h versus 31 h, *p* < 0.001). Early oral intake did not increase the rate of complications and it was assumed to be safe in bariatric surgery.

Evidence for the safety of diet texture progression after the early postoperative period is rare and lacks consensus with authors proposing a pureed diet period from 14 days to 2 months postoperatively (24, 25). Several postoperative protocols have been proposed, most of them as part of an enhanced recovery after surgery (ERAS) program (26–28). The ERAS guidelines for bariatric surgery do not report any texture progression timeline after surgery. A pureed diet is frequently put forward by bariatric surgeons for a period that they decide to establish with no clear evidence. That is why our practice changed progressively over time between 2015 and 2020. We compared 3 different recommendations and their impact on patient outcomes. With more than a hundred patients in each group, we reported similar complications rates at 90 days, comparable with international benchmarks of 9% for any complications at 30 days and 5% for severe complications (18, 29). Severe complications rates (Clavien-Dindo > grade 3a) and mortality were also comparable between the 3 groups. There was no primary leakage from the gastrojejunal anastomosis that might suggest any pressure from the food bolus. A slightly higher readmission rate in the short pureed diet group (6.3%) compared to the other groups (4 and 4.7% in groups 1 and 2 respectively) was found. However, this difference was not statistically significant and could be explained by a reduced length of stay in the short pureed diet group. Our findings support an early postoperative feeding and a pureed diet for no longer than 1 week, given the lack of increased morbidity and mortality at 90 days.

Hiatal hernia reconstruction during LRGYB was associated with a higher rate of severe complications in our series. The addition of an extra step during surgery may have participated in increasing the complexity of the procedure and its duration. However, the association between hiatoplasty and severe complications was not confirmed in the multivariate analysis. The same diet recommendation is used at our institution after anti-reflux surgery with 1 week of pureed diet. The same short period–pureed diet could be proposed to patients who underwent LRGYB with a hiatal hernia repair.

Unnecessary diet restriction could well impact patient quality of life. The positive psychological impact of feeding after surgery may have an important role in the recovery process. A short pureed diet does not seem to increase the number of emergency or specialized consultations. The shortening of a pureed diet period could be proposed without any closer postoperative monitoring. In the current movement of enhanced recovery, every patient care based on the surgeon’s beliefs are replaced by evidence-based practice in the perioperative care. The length of the pureed diet period is presently left to the surgeon’s discretion. However, in our experience, this recommendation could be safely standardized to a 1-week period.

And yet, our study has several limitations. First, the retrospective nature of the study implies a selection bias. The 3 groups were almost homogeneous except for BMI and diabetes type II. The BMI was higher in the 1-week pureed diet group and type II diabetes was lower in this group. This could have introduced a bias. However, there was still no difference in complication rates when we adjusted patients with a BMI > 40, as well as patients with diabetes mellitus in our multivariate analysis. The absence of difference between groups could be explained by a type II error. A similar study with a larger sample or a prospective design are necessary to validate our results.

A change in surgical technique occurred during the same period from mechanical anastomosis in the first period to hand-sewn anastomosis in the last period. Such technical changes could have introduced another bias. We included this parameter in the multivariate analysis, and we did not find any influence of the surgical technique on results.

Finally, we could have underestimated the rate of unplanned consultations as we recorded the consultations at our institution. Consultations at other places (family doctors, medical permanence) were not recorded. However, we reviewed all planned consultations for any mention of consultations outside of our institution and included them as an unplanned consultation.

## Conclusions

A regular diet could be introduced earlier in the post-LRYGB period than suggested by existing expert recommendations. Patients could have an early feeding with only 1 week of pureed diet, followed by a regular diet, without any increase in the postoperative complication rate or unplanned consultations. This may well contribute to patient comfort and recovery. Further prospective studies are required to validate such results and particular attention should be paid to patient quality of life.
